# Microstructure and Mechanical Properties of Unidirectional, Laminated C*_f_*/SiC Composites with α-Al_2_O_3_ Nanoparticles as Filler

**DOI:** 10.3390/nano12193406

**Published:** 2022-09-28

**Authors:** Lixia Yang, Fei Wang, Jiahao Liao, Zhaofeng Chen, Zongde Kou

**Affiliations:** 1 College of Materials Science and Technology, Nanjing University of Aeronautics and Astronautics, 29 Yudao St., Nanjing 210016, China; 2 Herbert Gleiter Institute of Nanoscience, School of Materials Science and Engineering, Nanjing University of Science and Technology, Nanjing 210094, China

**Keywords:** ceramic matrix composite, C*_f_*/SiC composites, α-Al_2_O_3_ nanoparticle filler, mechanical properties

## Abstract

The effects of an α-Al_2_O_3_ nanoparticle filler in the SiC matrix on the mechanical properties and failure mechanism of the unidirectional, laminated carbon fiber-reinforced SiC composites were investigated in this work. First, α-Al_2_O_3_ nanoparticles were added to the carbon fiber bundles using a slurry impregnation method, and then the C*_f_*/SiC composite with an α-Al_2_O_3_ nanoparticle filler (C*_f_*/SiC-Al_2_O_3_) was fabricated using a precursor infiltration and pyrolysis method. The microstructure of the C*_f_*/SiC-Al_2_O_3_ composite showed chemical compatibility between the α-Al_2_O_3_ and the pyrolysis SiC. The C*_f_*/SiC-Al_2_O_3_ composite with a low porosity of ~6.67% achieved a good flexural strength of 629.3 MPa and a good fracture toughness of 25.2 MPa·m^1/2^. The interlaminar shear strength of the C*_f_*/SiC-Al_2_O_3_ composite was 11.7 MPa. The SiC-Al_2_O_3_ matrix also presented a considerable Young’s modulus of 138.2 ± 8.66 GPa and hardness of 10.3 ± 1.03 GPa. Further analysis indicated that the good mechanical properties with the addition of an α-Al_2_O3 filler were not only related to the dense matrix and the improvement of the mechanical properties of the matrix. They also originated from the thermal residual compressive stress in the SiC matrix close to the α-Al_2_O_3_ nanoparticles caused by the thermal expansion mismatch, which could reflect and close the cracks in the matrix. The findings of this study provide more methods for designing new composites exhibiting a good performance.

## 1. Introduction

Continuous carbon fiber-reinforced silicon carbide matrix composites (C*_f_*/SiC) have been widely studied due to their low density, high specific strength, high hardness, nonbrittle mechanical behavior and good thermal shock tolerability. They are considered desirable high-temperature structural materials for advanced engines, gas turbines, thermal protection systems for space vehicles, components in scramjet and ramjet engines, etc. [1,2,3,4]. Seeking ways to improve the mechanical properties and oxidization resistance of the composites before application is vital to ensure their reliability and performance during service. The mechanical properties of C*_f_*/SiC composites can be improved by adding a reinforcing phase such as particles, fibers or whiskers into the SiC matrix [5]. Many research groups are putting their efforts into improving the mechanical properties of C*_f_*/SiC composites with a modified matrix using a second phase as a filler [6,7,8,9,10,11,12,13,14,15,16].

Zhu et al. [6] prepared 2D C*_f_*/SiC with a submicrometer SiC filler using a polymer infiltration and pyrolysis (PIP) process. With the addition of a SiC filler, the flexural strength of the C*_f_*/SiC composites increased from 120 MPa to 232 MPa when the content of the SiC filler increased from 0 to 40 wt.%. Jian et al. [8] investigated the mechanical properties of C*_f_*/SiC composites with SiC as an inactive filler prepared using polycarbosilane (PCS)/divinylbenzene pyrolysis. The results showed that the flexural strengths of the composites increased from 130 to 246.4 MPa when the content of the SiC filler increased to 30 wt.%. Shi et al. [9] reported that the addition of SiB_4_ to a SiC matrix could effectively increase the bulk density of the C*_f_*/SiC composites from 1.63 to 2.23 g·cm^−3^ and the flexural strength from 135 to 330 MPa. Cao et al. [10] investigated the mechanical properties of C*_f_*/SiC composites with a SiBC filler which showed a flexural strength of 276 MPa and elastic modulus of 78 GPa, as the reaction between the B_4_C and molten Si to form the SiC-SiBC matrix could fill the interbundle pores well, which improved the mechanical properties of the composite. Pi et al. [16] explored the effect of a ZrB_2_ and ZrC filler on the mechanical properties of C*_f_*/SiC composites prepared using a reactive melt infiltration method, which showed that with the addition of a ZrB_2_ and ZrC filler, the flexural strength increased from 350 ± 15 MPa to 380 ± 9 MPa. Galizia et al. [17] investigated the mechanical properties of C*_f_*/SiC composites with ZrB_2_ and TaC as an inactive filler prepared via a filament winding and PIP process. The results showed that 4 vol.% of ZrB_2_ and TaC did not affect the flexural strength of the commercial C*_f_*/SiC composite (SICARBON™ composite). These results suggest that the mechanical properties of C*_f_*/SiC composites could be effectively increased by the addition of appropriate fillers.

The mechanical properties of C*_f_*/SiC composites are determined by their microstructures. Density is the one of the most important factors [18] and in general, high density corresponds to high mechanical properties. Alpha-alumina (α-Al_2_O_3_) is a popular sintering additive for fabricating liquid phase sintered SiC ceramics [19,20]. The use of α-Al_2_O_3_ is supposed to promote the densification of SiC and thus result in higher fracture toughness compared with solid phase sintered SiC. However, there is a lack of research on the addition of α-Al_2_O_3_ as a sintering aid in the matrix of C*_f_*/SiC composites. In addition, the density of C*_f_*/SiC composites is also governed by the fabrication process [18]. The PIP process, which is most widely used to prepare C*_f_*/SiC, offers many potential advantages, such as a low processing temperature, controllable ceramic compositions and near-net shape ability. Despite these advantages, the basic limitation of the PIP process is the high volume shrinkage (about 50–60%) and pronounced porosity accompanied by the mass loss of the small molecules of the polymer during pyrolysis [8]. As a result, many infiltration–cure–pyrolysis cycles are required to densify the composites. To solve the problem, α-Al_2_O_3_ can be used as a filler to be added to the carbon fiber bundles before the fiber preform weaving step to improve the manufacturing efficiency of the matrix. In addition, α-Al_2_O_3_ can effectively reduce the diffusion rate of oxygen, which is also a favorable factor for serving in a high-temperature air environment [21]. Furthermore, α-Al_2_O_3_ can be used as a stress sensor using the detecting photoluminescence piezospectroscopy (PLPS) [22] of the persistent impurity of Cr^3+^ in α-Al_2_O_3_ to investigate thermal residual stress in the matrix of C*_f_*/SiC composites.

Therefore, in this work, α-Al_2_O_3_ nanoparticles were used as a sintering aid and a filler to enhance the mechanical properties of the C*_f_*/SiC composite process, denoted as C*_f_*/SiC-Al_2_O_3_. First, the α-Al_2_O_3_ nanoparticles were added to the carbon fibers using a slurry impregnation method, and then the SiC matrix was prepared using a PIP and vacuum pressure infiltration process. Finally, the phases, microstructure and mechanical properties of the C*_f_*/SiC-Al_2_O_3_ composites were investigated, and the enhancing mechanisms were analyzed and discussed.

## 2. Materials and Methods

### 2.1. Preparation of Materials

T800 carbon fibers (Toray Co., Tokyo, Japan), α-Al_2_O_3_ nanoparticles (Ningbo Jinlei Nano Material Technology Co., Ningbo, China) and PCS (Tunacera Materials Co., Suzhou, China) were used as raw materials. PCS is the precursor of SiC, with a ceramic yield of 60 wt.%. The employment of high strength T800 carbon fibers is beneficial to further improve the mechanical properties of composites. The properties of the T800 carbon fibers and α-Al_2_O_3_ nanoparticles are listed in Table 1 and Table 2, respectively.

**Figure 1:**
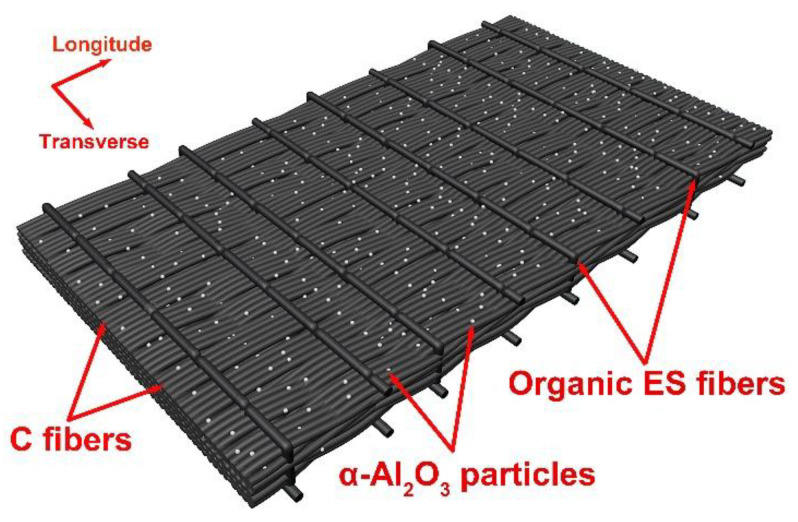
Schematic diagram of unidirectional, laminated T800 carbon fibers preforms.

The reinforcement of the C*_f_*/SiC-Al_2_O_3_ composite was unidirectional, laminated T800 carbon fiber preform which had a volume fraction of about 40%. The approximate 40% volume fraction was calculated based on the ratio of the bulk density of the carbon fiber preforms to the density of the carbon fibers. Before fiber preform weaving process, α-Al_2_O_3_ nanoparticles with a volume fraction of about 14% were added to the carbon fiber bundles using a slurry impregnation method [9]. The volume fraction of α-Al_2_O_3_, *Vol_A_* (%) was determined by Equation (1):

(1) $$Vol_{A}=\frac{m_{CP}-m_{C}}{\rho_{A}\cdot V_{CP}}\times 100\%$$

where *Vol_A_* is the volume fraction of α-Al_2_O_3_ in the C preforms; *m_CP_* is the weight of C fiber preforms; *m_C_* is the weight of C fibers; *ρ_A_* is the density of α-Al_2_O_3_ particles, and *V_CP_* is the volume of carbon fiber preforms. Then the volume fraction of α-Al_2_O_3_ was determined to be 14% by Equation (1) in the C preforms. First, the α-Al_2_O_3_ slurries were prepared using high-energy ball milling by dispersing α-Al_2_O_3_ nanoparticles in ethanol [23,24]. Then, the carbon fibers were infiltrated with alumina powder slurries and dried in a vacuum freeze drier [9]. Schematic diagram of unidirectional, laminated carbon fiber preforms with the addition of α-Al_2_O_3_ nanoparticles is shown in Figure 1. Note that the longitudinal carbon fibers were fixed in the fabric using transverse organic ES fibers. Before densifying the composites, carbon fiber preforms were coated with 0.3 μm thickness pyrolytic carbon (PyC) interphase under 1100 °C and 500 Pa, where argon and propylene were used as carrier gas and carbon source, respectively. The thicknesses of the PyC coating were examined using an image analysis method with ImageJ software on SEM images of polished sections. The average thickness was determined by the cross-sectional area of PyC coating divided by the length of interface. Each average thickness datum was from at least 20 measurements.

**Figure 2:**
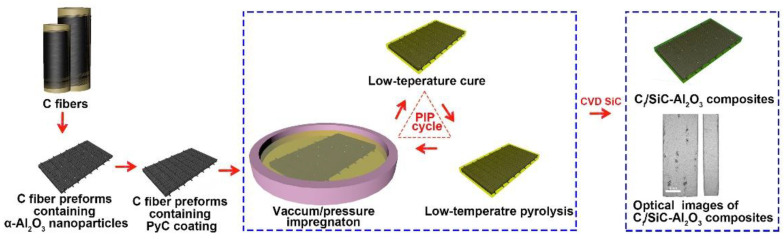
Schematic diagram of the preparation process of the C*_f_*/SiC-Al_2_O_3_ composite.

The C*_f_*/SiC-Al_2_O_3_ composite was prepared using the PIP method [25,26]. The schematic diagram of the PIP process is shown in Figure 2. In Figure 2, the dimensions of optical images of C*_f_*/SiC-Al_2_O_3_ composites after deposition are shown. A 50 wt.% PCS/xylene solution was used as the precursor. The detailed steps are as follows: firstly, the preforms were dipped into the solution under pressure for more than 3 h to improve the impregnation efficiency; after impregnation, the preforms were dried at 150 °C for 3 h in a vacuum drying oven and were pyrolyzed at 1100 °C for 1 h in Ar atmosphere in tube furnace; the impregnating–drying–pyrolyzing process was performed 16 times; finally, the pyrolyzed green composites were annealed at 1300 °C for 3 h in an Ar atmosphere for densification. 

After PIP process, the SiC overcoating was deposited using CVD method [27], where methyltrichlorosilane (MTS), hydrogen and argon were used as the original SiC source, carrier gas and protective gas, respectively. The flow rate of MTS, hydrogen and argon were fixed at 15, 150 and 150 sccm, respectively. The deposition was carried out at 1300 °C for 3 h under a total pressure of 600 Pa. 

### 2.2. Phase and Microstructure Characterization

The phases of the matrix in the C*_f_*/SiC-Al_2_O_3_ composite were identified using X-ray diffraction (XRD; Ultima IV, Rigaku, Japan). Note that the matrix of the composites was ground into powders for XRD examination. The XRD pattern was collected in the 2θ range of 10°-80° with a step size of 0.067° and a counting time of 1 s for each step. Scanning electron microscopy (SEM; TESCAN LYRA3 GMH, Brno, Czech) was employed for observing the morphology of the polished cross-sectional and fracture region of the composites. Chemical analysis of the composites was executed using energy dispersive X-ray spectroscopy (EDS, Oxford Aztec, X-Max50). Selected area electron diffraction (SAED) in transmission electron microscopy (TEM; Talos F200S G2, Hillsboro, OR, USA) was employed to further determine the morphology and crystal structures of the matrix in the composites [28].

### 2.3. Mechanical Property Tests

The bulk density and open porosity of the C*_f_*/SiC-Al_2_O_3_ composite were measured using an Archimedes method. The bulk density, *ρ (g·cm^−^^3^)* and open porosity, *P_a_* (%) were determined by Equations (2) and (3) [29], respectively:

(2) $$\rho =\frac{m_{3}\cdot D_{t}}{m_{3}-m_{2}}$$

(3) $$P_{a}=\frac{m_{3}-m_{1}}{m_{3}-m_{2}}\times 100\%$$

where *ρ* is the bulk density, *P_a_* is the open porosity, *m*_1_ is the dry weight, *m*_3_ is the saturated weight, and *D*_t_ is the density of the saturating liquid at room temperature. *m*_2_ is the Archimedes weight, which is the weight of the saturated specimen suspended in a container of the saturating liquid. Three-point bending tests were carried out using an electronic universal testing machine (CMT4503, SANS Co. Ltd., Shenzhen, China) at ambient temperature (25 °C). According to the standards in ASTM C1341-16, the sample geometry of three-point bending tests was 60 mm × 9 mm × 4 mm, the support span was 40 mm, and the cross-head speed was 0.5 mm/min. The interlaminar shear strength, *ILSS* (MPa) was determined by Equation (4) [17]:

(4) ILSS=3P/4wt

where *ILSS* is the interlaminar shear strength, *P* is the maximum applied force of three-point bending tests, and *w* and *t* are average measured width and thickness of the three-point bending specimen, respectively. The fracture toughness was determined by the single-edge notched beam (SENB) method [30]. According to the standards in ASTM C1421, the sample geometry of SENB samples with a notch depth of around 1.5 mm was 40 mm × 4 mm × 3 mm, with the span of 30 mm and a crosshead speed of 0.05 mm/min under three-point bending. Three samples were used for three-point bending tests. 

### 2.4. Residual Stress Measurement Using Photoluminescence Piezospectroscopy

The residual stresses in the α-Al_2_O_3_ particles for the C*_f_*/SiC-Al_2_O_3_ composites were evaluated using photoluminescence piezospectroscopy (PLPS) [25,31] with a Raman microscope (LabRAM HR Evolution; Horiba, France) coupled with a green laser (wavelength = 532 nm). Twenty measurements were used for residual stress tests.

### 2.5. Young’s Modulus and Hardness of the Matrix Using Nano-Indentation

The Young’s modulus and hardness of the matrix for the C*_f_*/SiC-Al_2_O_3_ composites were also detected using a nano-indentation instrument (Micro Materials Ltd., Camarillo, CA, USA) with Berkovich tip. The indentation measurements were performed on the cross-sections. At least six indentations were measured in the matrix using a force of 400 mN with a loading rate of 40 mN/s [32]. The testing parameters were based on the power-law fit method by Oliver and Pharr in 1992 [33], which was optimized in 2004 [34]. The hardness, *H* (GPa) and Young’s modulus, *E* (GPa) were determined by Equations (5) and (6) [35], respectively:

(5) $$H=\frac{P_{\mathrm{Max}}}{A_{r}}$$

(6) $$\frac{1}{E_{r}}=\frac{\left(1-\nu_{i}^{2}\right)}{E_{i}}+\frac{\left(1-\nu_{s}^{2}\right)}{E_{s}}$$

where *P*_Max_ is the maximum load of nano-indentation measurement, *A*_r_ is the vertical projected area of the contact surface, and *ν* is the Poisson’s ratio. *E*_r_ is the reduced modulus. *E*_i_ and *E*_s_ are Young’s modulus of the indenter and the composite, respectively. As the Berkovich tip is a diamond indenter tip, the values of *E*_i_ and *ν*_i_ are 1141 GPa and 0.07, respectively. The Poisson’s ratio of the SiC matrix is 0.17 [36]. 

## 3. Results

### 3.1. Phase Characterization of the Matrix

**Figure 3:**
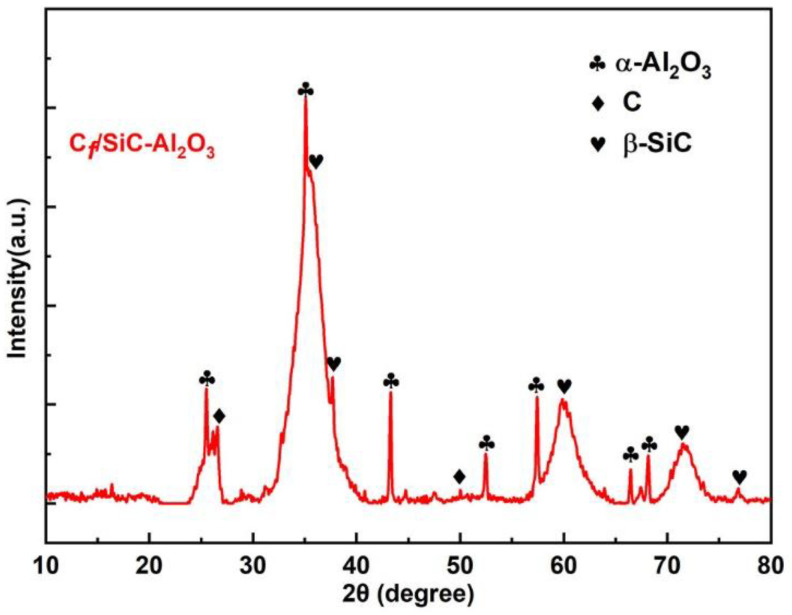
XRD patterns of the matrix for the C*_f_*/SiC-Al_2_O_3_ composite in a range of 2θ between 10° and 80°.

**Figure 4:**
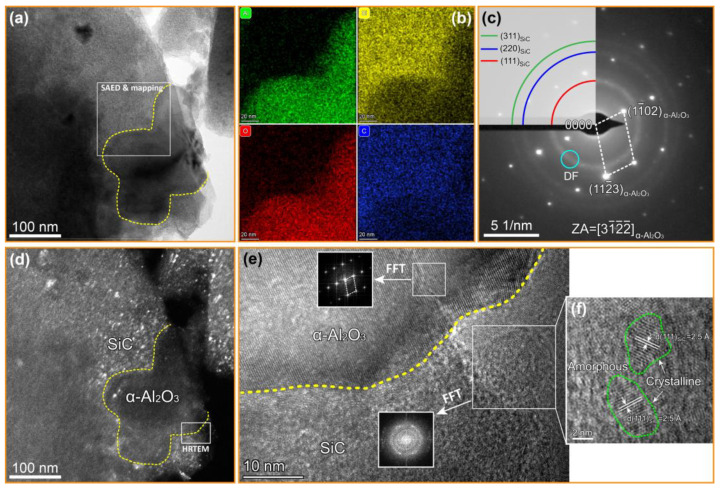
(**a**) Bright field TEM image of the SiC matrix with α-Al_2_O_3_ nanoparticles of the C*_f_*/SiC-Al_2_O_3_ composite; (**b**) elemental mapping images of Al, Si, O and C of boxed area in (**a**); (**c**) SAED pattern of boxed area in (**a**); (**d**) dark-field TEM image of (**a**), obtained using the circled reflection in (**c**); (**e**) HRTEM images of the framed region in (**d**); and (**f**) enlarged view of the boxed SiC area in (**d**).

Figure 3 shows the XRD patterns of the matrix from the C*_f_*/SiC-Al_2_O_3_ composite in a range of 2θ between 10° and 80°. The XRD pattern indicates that the matrix consists of C, β-SiC and α-Al_2_O_3_, where C and β-SiC are the pyrolytic products of the PCS (i.e., pyrolytic β-SiC). The XRD peaks of the pyrolytic β-SiC show a broad full width at half maximums (FWHMs) due to the nanosized β-SiC crystallites growing within an amorphous mass, as confirmed by further analysis in Figure 4. 

Figure 4a shows the bright field (BF) TEM image of the matrix in the C*_f_*/SiC-Al_2_O_3_ composites, where an α-Al_2_O_3_ particle with the size of ~180 nm is outlined by the yellow dotted line. Figure 4b shows the elemental mapping images of Al, Si, O and C of the boxed area in Figure 4a. It is clear that the Al and O elements are distributed in the outlined region, while the Si and C elements are mainly distributed complementary to Al and O. It is thus speculated that the particle outlined by the yellow dotted line is α-Al_2_O_3_, which is embedded into the SiC matrix. The SAED patterns of the boxed area in Figure 4a were obtained to confirm the phase composition, as shown in Figure 4c. The diffraction rings in Figure 4c were identified as SiC; the periodic diffraction spots were identified as the α-Al_2_O_3_ phase along the $\left[3\bar{1}\bar{1}\bar{2}\right]$ zone axis. To observe the morphological features of the SiC matrix, the TEM dark-field (DF) images are also presented in Figure 4d, which shows a large number of SiC nanocrystalline particles (bright spots). The high-resolution transmission electron microscope (HRTEM) image of the framed region in Figure 4d is shown in Figure 4e, which displays a well-bonded interface between α-Al_2_O_3_ and SiC in the matrix. A region in α-Al_2_O_3_ and SiC was selected, respectively, to perform fast Fourier transform (FFT). It can be seen that α-Al_2_O_3_ exhibits good crystalline characteristics, while SiC exhibits the characteristics of the coexistence of crystalline and amorphous states, mainly due to the nanosized β-SiC crystallites growing within an amorphous mass. An enlarged view of the framed SiC region is given in Figure 4f, from which it can be seen that the SiC nanocrystals are dispersed in the amorphous structure, and this morphological feature is in good agreement with the SAED patterns and DF image. In addition, no obvious second phase formed on the Al_2_O_3_ particle/SiC matrix interface. Both the XRD and TEM results suggest that the α-Al_2_O_3_ nanoparticles did not react with the pyrolysis products or the SiC precursor (PCS) during the PIP process for the C*_f_*/SiC-Al_2_O_3_ composites.

### 3.2. Microstructure Characterization

**Figure 5:**
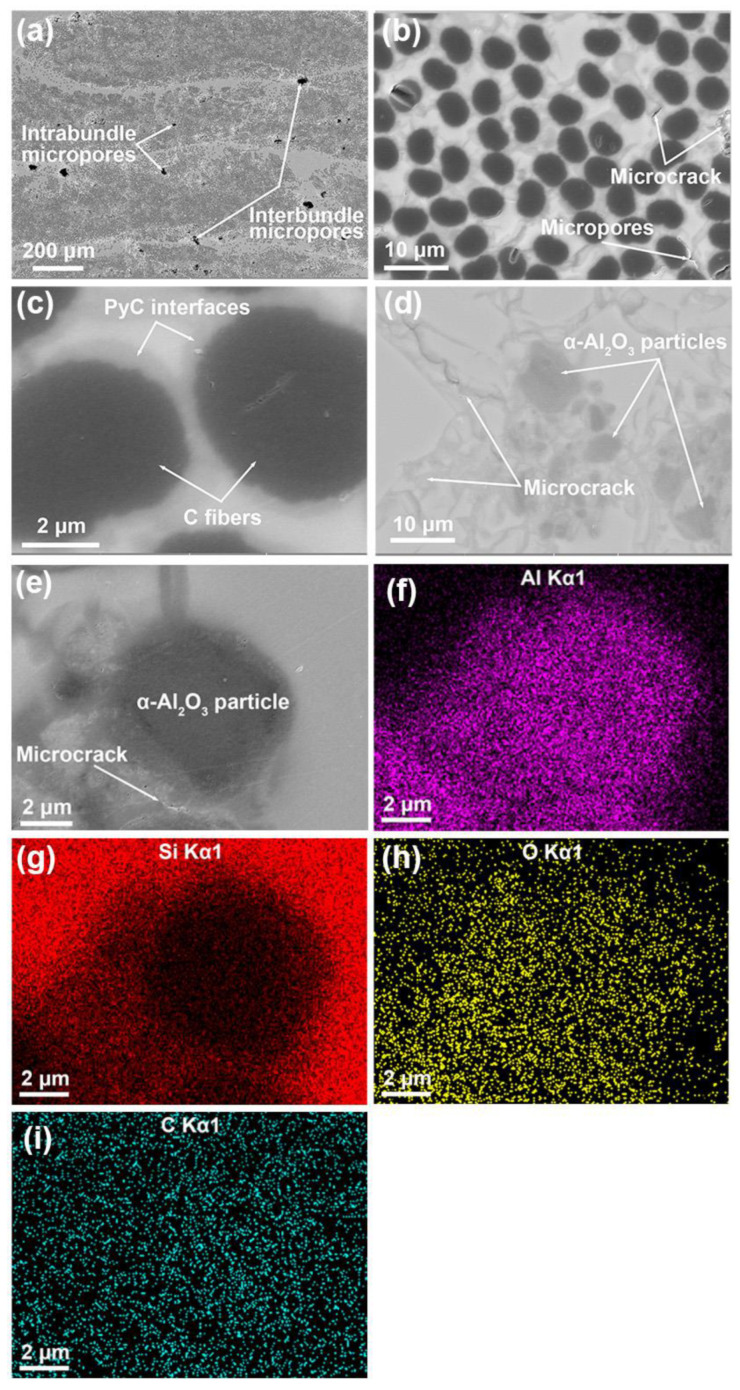
(**a**–**e**) Cross-sectional backscattered SEM images of the C*_f_*/SiC-Al_2_O_3_ composite and (**f**–**i**) EDS maps of the (**e**).

Figure 5a–d show the polished cross-sectional backscattered SEM images of the C*_f_*/SiC-Al_2_O_3_ composite. The darker parts in Figure 5a–c are carbon fibers and pores, while the brighter parts are the matrix. The carbon fibers are vertically aligned with the composite cross-section and inhomogeneously distributed in the composite. The intrabundle and interbundle gaps are sufficiently filled with the high dense matrix, confirming the effective infiltration and densification process. Only a few micropores can be observed in the matrix. These micropores were induced by the disruption around the fibers during the vacuum and pressure infiltration, which is referred to as the ‘wall effect’ during matrix densification [25,37,38]. 

Figure 5d shows the enlarged views of the matrix in the C*_f_*/SiC-Al_2_O_3_ composite. A small number of randomly distributed gray particles can be observed in the SiC matrix. The EDS maps in Figure 5e–i show that the gray particles are rich with Al and O, indicating that the gray particles are α-Al_2_O_3_ with particle sizes of about 1~10 μm. These observed microsized α-Al_2_O_3_ particles are a result of the broad size distribution of the particles. 

**Figure 6:**
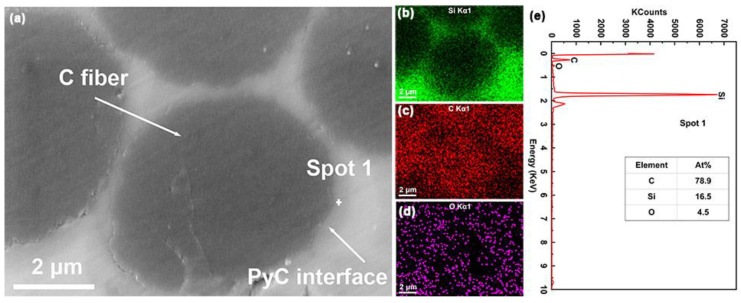
(**a**) Cross-sectional backscattered SEM micrographs of the C*_f_*/SiC-Al_2_O_3_ composite and (**b**–**e**) corresponding EDS analysis.

Figure 6a shows the enlarged views of the intrabundle in the C*_f_*/SiC-Al_2_O_3_ composite. A layer of gray ring surrounds the C fiber. The EDS maps of the gray ring, as shown in Figure 6a–e, are mainly composed of the C element, which confirms that the gray ring is a PyC coating. From Figure 5b,c and Figure 6a, it can be seen that the PyC coating with a thickness of 250 ± 50 nm was well-bonded with the fibers and matrix as the interphase for the C*_f_*/SiC-Al_2_O_3_ composite. 

### 3.3. General Properties and Mechanical Properties

The bulk density and open porosity of the C*_f_*/SiC-Al_2_O_3_ composite are listed in Table 3. The bulk density of the C*_f_*/SiC-Al_2_O_3_ composites was 2.03 g·cm^−3^, while the mean open porosity of the C*_f_*/SiC-Al_2_O_3_ composite was 6.67%, which is lower than the commonly reported porosity (8–15%) of the C*_f_*/SiC composites prepared using PIP methods [39,40,41,42]. This result indicates that the combination of the slurry impregnation method and the PIP with the high pressure impregnation process is an effective method to achieve a relatively lower open porosity [43] and that the addition of α-Al_2_O_3_ particles could promote densification of the SiC matrix during sintering.

**Figure 7:**
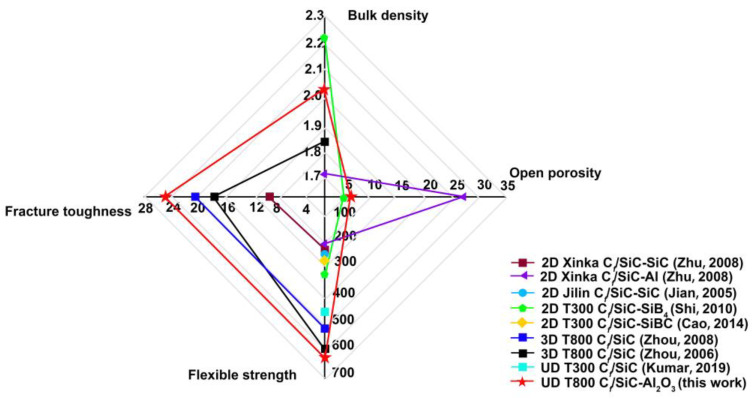
Comparison of general properties and mechanical properties between C*_f_*/SiC-Al_2_O_3_ composites (this work) and other C*_f_*/SiC composites in previous works [6,7,8,9,10,11,12,13]. (UD refers to the unidirectional structure).

The mechanical properties of the C*_f_*/SiC-Al_2_O_3_ composite are summarized in Table 4. The flexural strength and fracture toughness of the C*_f_*/SiC-Al_2_O_3_ composite was 629.3 MPa, and the values were 25.2 MPa·m^1/2^, respectively. The general and mechanical properties of other C*_f_*/SiC composites in previous works [6,7,8,9,10,11,12,13] are listed in Table 4. Figure 7 shows a comparison of the general properties and mechanical properties between the C*_f_*/SiC-Al_2_O_3_ composite (this work) and the C*_f_*/SiC composites in previous works [6,7,8,9,10,11,12,13]. The flexural strength and fracture toughness of the C*_f_*/SiC-Al_2_O_3_ composite was higher than those of three-dimensional four-directional braided T800-HB C*_f_*/SiC composites with SiC interlayers [11], three-dimensional braided T800 C*_f_*/SiC composites [12], unidirectional T300 C*_f_*/SiC [13] and the other C*_f_*/SiC composites modified by Al [7], SiC [6,8], SiB_4_ [9] or SiBC [10]. This mainly resulted from the relatively dense and high mechanical properties of the matrix for the C*_f_*/SiC-Al_2_O_3_ composite after being densified with α-Al_2_O_3_ and the compressive stress field in the SiC matrix due to the thermal misfit, which will be discussed in Section 4.

The interlaminar shear strength of the C*_f_*/SiC-Al_2_O_3_ composite is listed in Table 5. The interlaminar shear strength of the C*_f_*/SiC-Al_2_O_3_ composite was 11.7 MPa. An overview of the material interlaminar shear strength data for the C*_f_*/SiC composite [44,45] is listed in Table 5. In Table 5, the interlaminar shear strength of the C*_f_*/SiC composites prepared using the CVI method is higher than the C*_f_*/SiC-Al_2_O_3_ composites, mainly due to the mechanical properties of the CVI SiC matrix are higher than those of the PIP [46]. The interlaminar shear strength of the C*_f_*/C-SiC prepared using the LSI method and the C*_f_*/SiC composites prepared using the LPI method manufactured by MAN is higher than the C*_f_*/SiC-Al_2_O_3_ composites. The interlaminar shear strength of the C*_f_*/SiC composites using the LPI method manufactured by Dornier is lower than the C*_f_*/SiC-Al_2_O_3_ composites. The interlaminar shear strength of the C*_f_*/SiC-Al_2_O_3_ composites is in the range of the interlaminar shear strength of the C*_f_*/SiC prepared using the PIP method.

**Figure 8:**
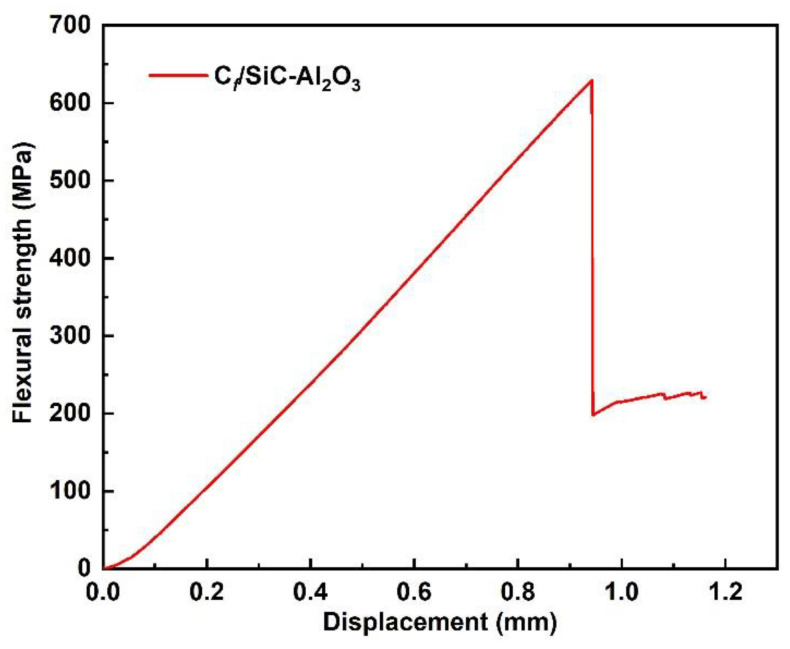
Typical flexural strength displacement curve of the C*_f_*/SiC-Al_2_O_3_ composite.

Figure 8 shows the typical flexural stress displacement curve of the C*_f_*/SiC-Al_2_O_3_ composite. The composite shows typical nonbrittle fracture behaviors in the three-point bending testing: the stress increases monotonously with displacement without early matrix cracking behavior; when the stresses reach the maximum values, the curve drops due to fiber bundle failure; subsequently, a step-down nonbrittle stage is observed, indicating energy dissipation during the crack propagation through toughening mechanisms such as crack deflection, fiber sliding and pullout before the composite ultimately ruptures. The total energy dissipation for the C*_f_*/SiC-Al_2_O_3_ composites by measuring the area under the flexural load displacement curve was 757.0 J.

**Figure 9:**
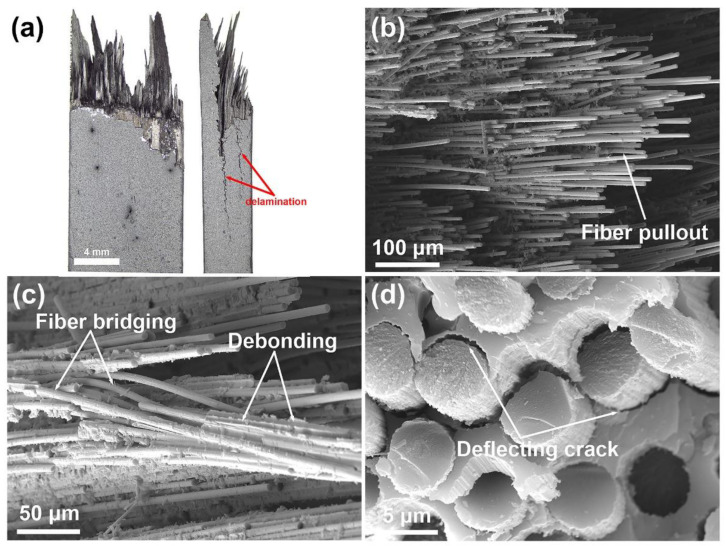
(**a**) Typical optical microscope images of the fracture morphology of the C*_f_*/SiC–Al_2_O_3_ composite; and (**b**–**d**) secondary electron SEM images of the fracture morphology of the C*_f_*/SiC–Al_2_O_3_ composite after bending test.

Figure 9a shows typical optical microscope images of the fracture morphology for the C*_f_*/SiC-Al_2_O_3_ composite after the bending test. The fracture surfaces of the C*_f_*/SiC-Al_2_O_3_ composite are ‘brushy’ and show extensive long fiber pullout, with the fiber pullout length reaching ~6 mm. The composite after the bending test exhibits delamination behavior, which is mainly due to the poor interlaminar shear strength of the unidirectional, laminated structure. Delamination behavior as a way to dissipate fracture energy can improve failure tolerance and fracture toughness of the composites [24]. The delamination behavior of composites contributes to the step-down characteristic of the nonbrittle stage in Figure 8. Figure 9b–d show the SEM images of the fracture morphology of the C*_f_*/SiC-Al_2_O_3_ composite after the bending test. A considerable number of fiber monofilament pullouts and fiber bundle pullouts are clearly discernible. Irregularly shaped matrix chips are retained on the pulled-out fibers, which play a role in transmitting load. Moreover, fiber bridging and deflection crack failure modes are also observed in Figure 9c,d. These failure modes for the composites provide energy dissipation which leads to an increase in fracture toughness and strength [47,48,49,50].

### 3.4. Young’s Modulus and Hardness of the Matrix

**Figure 10:**
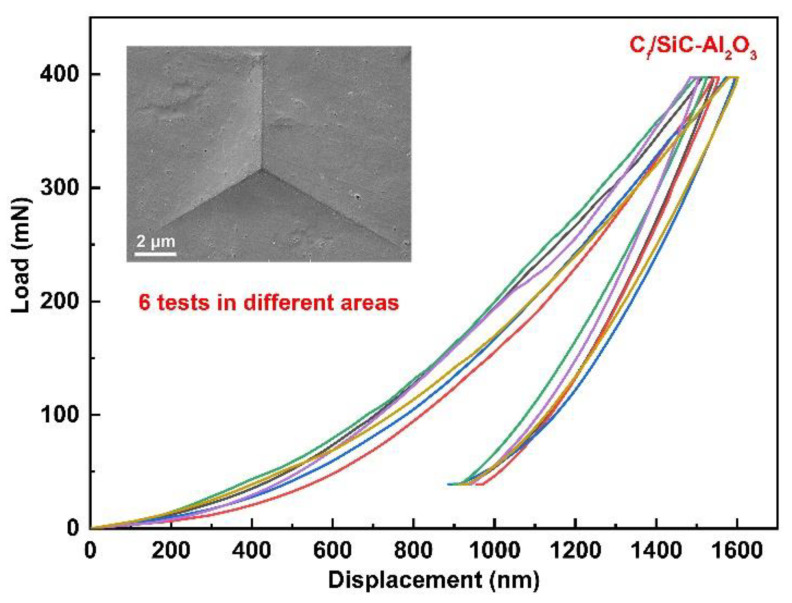
Load displacement curves of the SiC matrix for the C*_f_*/SiC-Al_2_O_3_ composites under a maximum indenting load of 400 mN.

The Young’s modulus and hardness of the SiC-Al_2_O_3_ matrix were measured using nano-indentation. Figure 10 shows the load displacement curves of the matrix for the C*_f_*/SiC-Al_2_O_3_ composites under a maximum indenting load of 400 mN. The load displacement curve is smooth without pop-in behavior [51]. The nano-indentation of Young’s modulus and hardness of the C*_f_*/SiC-Al_2_O_3_ composites are listed in Table 6. The measured average Young’s modulus and hardness of the nano α-Al_2_O_3_ modified SiC matrix were 138.2 ± 8.66 and 10.3 ± 1.03 GPa, respectively. These are clearly higher than those of the pyrolytic β-SiC matrix in previous works, which range from 74 to 126 GPa and 6 to 9.58, respectively [52,53,54]. The higher Young’s modulus and hardness of the matrix facilitate higher breaking strength for composites [55].

## 4. Discussion

As shown in Figure 7 and Figure 10, the C*_f_*/SiC-Al_2_O_3_ composite shows excellent mechanical properties (flexural strength and fracture toughness) and considerable mechanical properties of the matrix (Young’s modulus and hardness) compared with previous works [6,7,8,9,10,11,12,13,52,53,54]. The good mechanical properties of the SiC-Al_2_O_3_ matrix could be related to the relatively dense matrix using nano α-Al_2_O_3_ as a filler, which generally acts as a sintering aid for SiC to improve the densification of the matrix [56]. A denser and higher strength matrix plays a key role in transferring stress. During loading, stress is effectively transferred from the dense matrix to the PyC interphase and the fibers. Matrix cracks propagating in the dense SiC-Al_2_O_3_ matrix can be deflected in the PyC interphase, leading to the pullout of the fibers [9]. As the α-Al_2_O_3_ nanoparticle sintering aid will also improve the bonding in the matrix, early matrix cracking was not observed on the flexural stress versus the displacement curves (Figure 8). This finally decreased the chances of microcrack formation in the matrix [10]. As a result, the C*_f_*/SiC-Al_2_O_3_ composites showed improved mechanical properties.

In addition, in searching the factors that contribute to the enhancing mechanism of the C*_f_*/SiC-Al_2_O_3_ composite, it was found that residual compressive stress fields generated in the matrix due to the thermal misfit could deflect or close cracks and improve the mechanical properties of the composite. 

**Figure 11:**
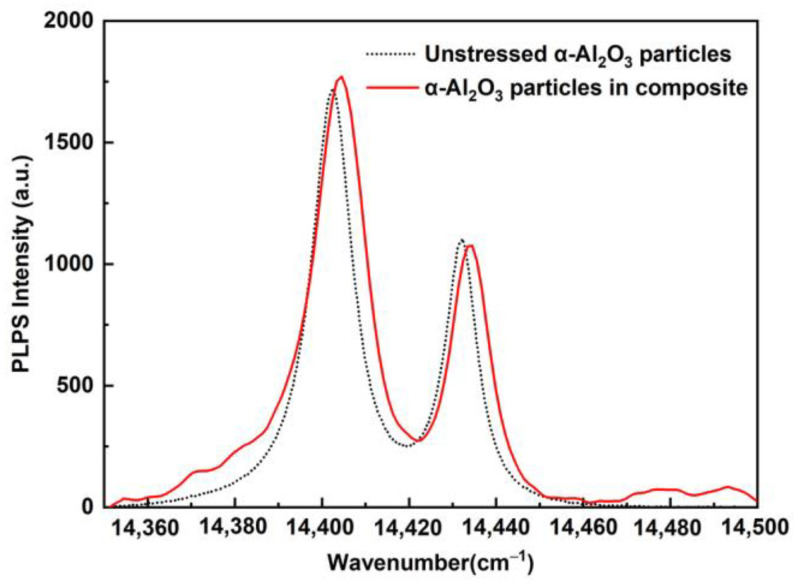
Typical PLPS spectra of Cr^3+^ R_1_ and R_2_ peaks from unstressed α-Al_2_O_3_ particles and the α-Al_2_O_3_ particles in C*_f_*/SiC-Al_2_O_3_ composite.

Due to the thermal expansion mismatch between the α-Al_2_O_3_ nanoparticles and pyrolytic β-SiC, thermal residual stresses will generate in the α-Al_2_O_3_ nanoparticles and the surrounding matrix. The residual stresses in the α-Al_2_O_3_ nanoparticles can be measured using PLPS [22,24,25]. Figure 11 shows the typical PLPS spectra of Cr^3+^ R_1_ and R_2_ peaks from unstressed α-Al_2_O_3_ particles and the α-Al_2_O_3_ nanoparticles in the C*_f_*/SiC–Al_2_O_3_ composite. Twenty points were measured for the C*_f_*/SiC-Al_2_O_3_ composite. The residual stress (*σ*) can be obtained from the peak shift (Δ*P*) between the α-Al_2_O_3_ nanoparticles in the C*_f_*/SiC-Al_2_O_3_ composite and the unstressed α-Al_2_O_3_ particles as follows [24]:

(7) $$\sigma =\frac{\Delta P}{2\Pi_{a}+\Pi_{c}}$$

where $\Pi_{a}$ and $\Pi_{c}$ are the piezospectroscopy coefficients of the crystallographic a and c axis of the Cr^3+^-doped Al_2_O_3_; and the sum of the piezospectroscopy coefficients (2$\Pi_{a}$ + $\Pi_{c}$) are 7.59 and 7.61 cm^−1^ GPa^−1^ for the R_1_ and R_2_ lines, respectively, as determined by He and Clarke [57]. Δ*P* is the R peak shift relative to the unstressed position *P*_0_, which is determined from the unstressed α-Al_2_O_3_ particles, as shown in Figure 11. The R_2_ peak shift is used for stress measurement as its peak shift maintains a linear relationship with the residual stress [58]. From Equation (7), the relationship between the residual stress (*σ*) in the α-Al_2_O_3_ nanoparticles for the composite and the R_2_ peak shift (Δ*P*_2_) can be expressed as follows:

(8) $$\sigma =\frac{\Delta P_{2}}{7.61}$$

The residual stresses in the α-Al_2_O_3_ nanoparticles of the C*_f_*/SiC-Al_2_O_3_ composite were determined to be 378.9 ± 85.8 MPa by Equation (8). The positive sign indicates that the stress is tensile stress, which mainly comes from the thermal expansion coefficient mismatch between the α-Al_2_O_3_ filler and the pyrolytic β-SiC matrix. Due to the force balance, compressive stresses will be generated in the matrix in the composites. 

Due to the limit stress influence ranges of the reinforcement phase (α-Al_2_O_3_ nanoparticles), for simplicity, the thermal residual stresses in the α-Al_2_O_3_ nanoparticles of the C*_f_*/SiC-Al_2_O_3_ composite ($\sigma_{{\mathrm{Al}}_{2}O_{3}}$) are theoretically calculated by [24,59]:

(9) $$\sigma_{{\mathrm{Al}}_{2}O_{3}}=\frac{E_{\mathrm{SiC}}E_{{\mathrm{Al}}_{2}O_{3}}(\alpha_{\mathrm{SiC}}-\alpha_{{\mathrm{Al}}_{2}O_{3}})\Delta T}{E_{{\mathrm{Al}}_{2}O_{3}}(1+\nu_{\mathrm{SiC}})+E_{\mathrm{SiC}}(1-\nu_{{\mathrm{Al}}_{2}O_{3}})}$$

where *E*, *α* and *ν* are the elastic modulus, CTE and Poisson’s ratio, respectively, and Δ*T* is the temperature difference. The subscripts ‘SiC’ and ‘Al_2_O_3_’ refer to the SiC matrix and the α-Al_2_O_3_ filler, respectively. The CTEs of α-Al_2_O_3_ and pyrolytic β-SiC are 8.0 × 10^−6^·K^−1^ [60] and 4.6 × 10^−6^·K^−1^ [61], respectively, for the temperature range 20–1000 °C. The values of *E* for α-Al_2_O_3_ and pyrolytic β-SiC are 400 GPa [60] and 138.2 GPa which are measured by nano-indentation, respectively. Poisson’s ratios for α-Al_2_O_3_ and pyrolytic β-SiC are 0.23 [60] and 0.17 [36], respectively. Accordingly, the average theoretical thermal mismatch stresses in the α-Al_2_O_3_ nanoparticles are calculated to be 417.19 MPa by Equation (9). The theoretical tensile stresses in the α-Al_2_O_3_ nanoparticles are slightly higher than the experimental value, which could be related to the fact that the amorphous products of the pyrolysis of PCS and the carbon fibers have not been considered in Equation (9). The thermal residual stresses in the α-Al_2_O_3_ nanoparticles filler or the SiC matrix are a key factor for affecting the composite property [61,62,63].

**Figure 12:**
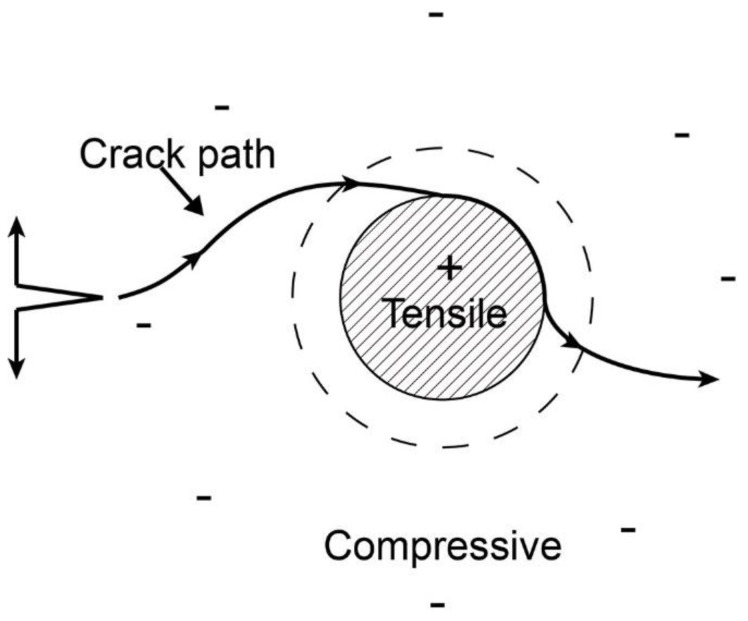
Toughening mechanism by thermal residual stress. Deflection of the crack by the particle and associated matrix stresses. The crack moving in the plane of the particle will first be deflected. As the crack moves around the particle, it can be attracted to the particle interface.

On the one hand, the SiC-Al_2_O_3_ matrix can be regarded as an α-Al_2_O_3_ particulate-reinforced SiC ceramic matrix composite, regardless of the carbon fibers temporarily. For the particulate-reinforced ceramic composite, toughening by thermal compressive residual stresses is one of the effective toughening mechanisms when the coefficient of the thermal expansion of the dispersed particles is larger than that of the matrix grains [63]. According to Wei [64] and Taya et al. [65], the thermal residual stress field in a particulate-reinforced ceramic composite consists of two regions: the tensile stress region in the particulates and the compressive stress region in the neighboring matrix, as shown schematically in Figure 12. Consider a semi-infinite crack surrounded by a particulate-reinforced ceramic matrix composite with a thermal residual stress distribution. The thermal residual compressive stress in the neighboring matrix can deflect the crack in the matrix to increase toughness, which is typically observed in most particulate-reinforced ceramic matrix composites [63]. In addition, the compressive residual stress in the neighboring matrix could result in the closing of the preparation processing-induced matrix microcracks. Consequently, the strength and toughness of the SiC ceramic matrix for the particulate-reinforced SiC ceramic matrix are improved by the thermal residual stress distribution around the particulates, which further contributes to the whole properties of the C*_f_*/SiC composite. 

On the other hand, for the whole carbon fiber-reinforced ceramic matrix composite system, thermal residual stress are also known to have a significant influence on the macroscopic mechanical behavior of CMCs by determining the stress states of the constituents in the composites and triggering damage to the composites (matrix cracking, fiber–matrix debonding) [61].

The radial thermal residual stress in the carbon fibers (*σ_f_*) due to thermal expansion mismatch can be expressed as:

(10) $$\sigma_{f}=\frac{E_{m}E_{f}(\alpha_{m}-\alpha_{f})\Delta T}{E_{f}(1+\nu_{m})+E_{m}(1-\nu_{f})}$$

where the subscripts ‘m’ and ‘f’ refer to the matrix and fibers, respectively. Since the radial CTE of carbon fiber (*α*_f_ = 10 × 10^−6^·K^−1^) is larger than that of the SiC matrix (*α*_SiC_ = 3.0 × 10^−6^·K^−1^), the carbon fiber is in a radial residual tensile stress state, and the SiC matrix is in a radial residual compressive stress state once cooled down from the processing temperature to room temperature. Since the axial CTE of the carbon fiber (*α*_f_ = 1.5 × 10^−6^·K^−1^) is lower than that of the SiC matrix (*α*_SiC_ = 3.0 × 10^−6^·K^−1^), the carbon fiber is in an axial residual compressive stress state, and the SiC matrix is in a radial residual tensile stress state once cooled down from the processing temperature to room temperature. The SiC matrix is still subjected to residual compressive stress due to the axial area of the C fibers being much smaller than the radial area. As the CTE of the two-phase matrix follows the simple rule of mixtures given by References [66,67], and the $\alpha_{{\mathrm{Al}}_{2}O_{3}}$ is equal to 8.0 × 10^−6^·K^−1^, the addition of an α-Al_2_O_3_ filler will result in a higher CTE of the matrix. According to Equation (10), a higher CTE of the matrix corresponds to a lower tensile stress in the fiber. Namely, the thermal compressive radial stress in the matrix decreases with the addition of the α-Al_2_O_3_ filler. For C*_f_*/SiC composites, thermal residual stress has a significant influence on the improvement of the properties of the composites. The less the thermal residual stress in the composites, the greater the first matrix cracking stress, and the more the whole properties of the composite can be promoted [61]. Zhu et al. [7] reported that the addition of a 20 wt.% Al filler to C*_f_*/SiC composites could effectively decrease the formation of microcracks in the SiC matrix. These results suggest that the matrix structure of C*_f_*/SiC composites could be effectively changed by the addition of fillers.

## 5. Conclusions

Unidirectional laminated C*_f_*/SiC-Al_2_O_3_ composites were fabricated using a PIP process. The phases, microstructure and mechanical properties of the composites were analyzed and discussed. The main findings are concluded as follows:

(1) The C*_f_*/SiC-Al_2_O_3_ composite had a good flexural strength of 629.3 MPa and fracture toughness of 25.2 MPa·m^1/2^ and exhibited a nonbrittle failure behavior. The interlaminar shear strength of the C*_f_*/SiC-Al_2_O_3_ composite was 11.7 MPa. The SiC-Al_2_O_3_ matrix also had a considerable Young’s modulus of 138.2 ± 8.66 GPa and hardness of 10.3 ± 1.03 GPa.
(2) The good mechanical properties of the C*_f_*/SiC-Al_2_O_3_ composites are related to its lower porosity (~6.67%), the good mechanical properties of the SiC-Al_2_O_3_ matrix and the thermal residual compressive stress in the matrix which can deflect or close cracks to improve the toughness and strength of the composite.
(3) The C*_f_*/SiC-Al_2_O_3_ composite showed excellent mechanical properties (flexural strength and fracture toughness) compared with conventional C*_f_*/SiC composites in previous works. This means the C*_f_*/SiC-Al_2_O_3_ composite could be used as an ideal high temperature structural material for advanced engines, gas turbines, spacecraft thermal protection systems, scramjets and ramjet components.
(4) Future work should be focused on the comparison of the mechanical and oxidation resistance properties of C*_f_*/SiC composites with and without Al_2_O_3_. In addition, the relationship between the mechanical properties and the volume fraction of the Al_2_O_3_ filler should be comprehensively investigated.

## Figures and Tables

**Table 1:** Properties of T800 carbon fibers.

Sample	Fiber Diameter (μm)	Density (g·cm^−3^)	Tensile Strength (GPa)	Elastic Modulus (GPa)	Elongation (%)
T800 carbon fibers	5	1.81	5.5	294	1.9

**Table 2:** Properties of α-Al_2_O_3_ nanoparticles.

Trademark	Purity (%)	Density (g·cm^−3^)	Specific Surface Area (m^2^·g^−1^)	Average Particle Size (nm)	Crystal Structure
α-Al_2_O_3_-W01	99.9	3.9	45.45	30	α-Al_2_O_3_

**Table 3:** General and mechanical properties of the C*_f_*/SiC-Al_2_O_3_ composite.

Sample	Bulk Density (g·cm^−3^)	Open Porosity (%)	Flexible Strength (MPa)	Fracture Toughness (MPa·m^1/2^)	Interlaminar Shear Strength (MPa)
C*_f_*/SiC-Al_2_O_3_	2.03	6.67	629.3	25.2	11.7

**Table 4:** General and mechanical properties of other C*_f_*/SiC composites in previous works [6,7,8,9,10,11,12,13]. (UD refers to the unidirectional structure.)

Materials	Structure	Bulk Density (g·cm^−3^)	Open Porosity (%)	Flexible Strength (MPa)	Fracture Toughness (MPa·m^1/2^)	Reference
Xinka C*_f_*/SiC-SiC	2D	-	-	232	10	[6]
Xinka C*_f_*/SiC-Al	2D	1.72 ± 0.03	27	211 ± 13	-	[7]
Jilin C*_f_*/SiC-SiC	2D	-	-	246.4	-	[8]
T300 C*_f_*/SiC-SiB_4_	2D	2.23	6	330	-	[9]
T300 C*_f_*/SiC-SiBC	2D	-	-	276	-	[10]
T800 C*_f_*/SiC	3D	-	-	511.5	20.8	[11]
T800 C*_f_*/SiC	3D	1.86	-	600.8	18.5	[12]
T300 C*_f_*/SiC	UD	-	-	400–450	-	[13]

**Table 5:** Overview of material interlaminar shear strength data for the C*_f_*/SiC composites.

Property	Gasphase Infiltration (CVI) Process			Liquid Infiltration Process			Polymer Infiltration and Pyrolysis (PIP)	Polymer Infiltration and Pyrolysis (PIP)
	CVI (Isothermal)		CVI (p, T-Gradient)	Liquid Polymer Infiltration (LPI)		Infiltration (LSI)		
	C*_f_*/SiC	C*_f_*/SiC	C*_f_*/SiC	C*_f_*/SiC	C*_f_*/SiC	C*_f_*/C-SiC	C*_f_*/SiC	C*_f_*/SiC-Al_2_O_3_
Interlaminar shear strength (MPa)	26.1–46.8	35	45–48	10	35	28–33	10–12	11.7
Porosity (%)	10–15	10	10-15	10	15–20	2–5	10–25	6.67
Density (g/cm^3^)	2.0	2.1	2.1–2.2	1.8	1.7–1.8	1.9–2.0	1.6–1.8	2.03
Fiber preform	MWK-N	(0°/90°) PW	(0°/90°) PW	(0°/90°) PW	(0°/90°) PW	(0°/90°) PW	UD	UD
Manufacturer	NWPU	SNECMA	MAN	Dornier	MAN	DLR	-	-
Reference	[44]	[44]	[44]	[44]	[44]	[44]	[45]	This work

**Table 6:** The nano-indentation of Young’s modulus and hardness of the C*_f_*/SiC-Al_2_O_3_ composites.

Sample	Young’s Modulus (GPa)	Hardness (GPa)
C*_f_*/SiC–Al_2_O_3_	138.2 ± 8.66	10.3 ± 1.03

## Data Availability

Not applicable.
